# Online partner seeking and high-risk behaviour in men who have sex with men and male-to-female transgendered people in Mumbai, India: Implications for prevention strategies

**DOI:** 10.1371/journal.pone.0284602

**Published:** 2023-04-28

**Authors:** Shrikala Acharya, Vijay Karanjkar, Smita Chougule, Sachendra Katkar, Shashikant Patil, Vivek Dwivedi, Prashant Deshpande, Maninder Singh Setia

**Affiliations:** 1 Mumbai Districts AIDS Control Society, Mumbai, India; 2 Seth G S Medical College and KEM Hospital, Mumbai, India; 3 UW International Training and Education Center for Health, Mumbai, India; 4 Technical Support Unit, HLFPPT, Mumbai, India; 5 MGM Institute of Health Sciences, Mumbai, India; HJF: Henry M Jackson Foundation for the Advancement of Military Medicine Inc, NIGERIA

## Abstract

**Background:**

It is important to understand the current internet-related sexual behaviours of high-risk groups such as men who have sex with men (MSM). We designed the present study to understand the types of online/mobile apps used by MSM and male-to-female transgendered people/hijras [TGH] in Mumbai, India. We also compared the internet-related ‘partner seeking’ and ‘sexual behaviours’ in MSM and TGH in Mumbai, India.

**Methods:**

This is a cross-sectional analysis of secondary data collected (April to June 2020) from 8582 MSM and 4163 TGH from five targeted intervention programmes each in Mumbai, Maharashtra, India. Data on demographics, years of association with the intervention, number and type of online/mobile apps used, sexual behaviours including partners from virtual space and non-virtual (physical) space, group sex, attending parties, mobility for sexual partners, and HIV status were collected.

**Results:**

MSM were more likely to have mobile phone (88% vs 51%, p < 0.001) and internet access over the phone (78% vs 27%; p < 0.001) compared with TGH. The common apps used by MSM were Grindr (48%), Facebook (42%), and Blued (36%). MSM were more likely to have partners from virtual space (91% vs 67%; p < 0.001). A higher proportion of MSM had attended parties (28% vs 2%; p < 0.001), had group sex (16% vs 6%; p < 0.001), and were mobile for sex (25% vs 4%). MSM and TGH who had partners from virtual space were significantly more likely to report ‘missed a condom at least once during penetrative sex in the past one week’ (17% vs 12%; p<0.001). In HIV positive MSM, group sex, parties, and mobility for sex, were only in those who reported partners from the virtual space.

**Conclusions:**

Internet-based interventions for MSM should be incorporated in the existing targeted intervention programme and outreach workers should be trained in virtual outreach services. Among TGH, given the low reach and use of smartphones and apps, internet-based interventions may not be such a useful option, and the existing physical targeted intervention programmes should be strengthened.

## Introduction

With the rationalisation of cost and easy availability, internet penetration has increased in India. With an estimated 624 million current internet users, internet penetration in India has increased from 4% in 2007 to 50% in 2020 [1, 2]. In fact, a report in 2020 suggested that rural internet users surpassed urban internet users for the first time in India by end of 2019 [3]. Individuals in the age group of 20 to 39 years formed the largest user base, whereas those over the age of 40 years was least likely to use internet [4]. Over the past few years, many activities have moved online. These include banking facilities, payment facilities, booking of tickets to name a few. There has also been an increase in the number of websites and applications where individuals find sexual partners. Some of these are for the general population whereas others are targeted at specific groups (such as gay men, lesbians, and male-to-female transgendered people). Furthermore, though most of these sites advertise themselves only as friendship sites, few do exist which are sites for finding paid sexual partners explicitly.

Studies have found that 79% of men who have sex with men (MSM) use two or more virtual platform sites; they have an average of 3.7 virtual profiles. Furthermore, among these, about 74% also visited a physical space for finding a sexual partner [5]. Garga and co-workers found that individuals with more than one sexual partner, those who have high-risk behaviour, and those who have casual sex without discussion about sexually transmitted infections were more likely to use these dating apps [6]. Another study found that men who found their partners online were more likely to pay for sex compared with those who met partners at social venues [7]. A meta-analysis found that sexual encounters that were initiated online were more likely to have unprotected anal intercourse, whereas recent studies have found that app-users were more likely to have sexually transmitted infections (gonorrhea, chlamydia, and syphilis) [8–10]. In addition, though MSM who find their partners online are more likely to engage in risky behaviours, they are also more likely to have regular sexual risk assessments and treatment for sexually transmitted infections [11–13].

Thus, even though the internet is used by at-risk population to find sexual partners and engage with high-risk behaviour with these partners, potentially, the same internet can also be used to provide information, intervention, and services to these at-risk people [14, 15]. Hence, it is important to understand the current internet-related sexual behaviours of high-risk groups (key population). This knowledge will help us identify the gaps in the existing interventions and modifications needed for these outreach programmes. With this background, we designed the present study to understand the types of online/mobile apps used by MSM and male-to-female transgendered people [TGH] in Mumbai, India. We compared the internet-related ‘partner seeking’ and ‘sexual behaviours’ among MSM and TGH in addition to identifying the risk factors in both these groups in Mumbai, India.

## Methods

The present study is a cross-sectional analysis of the secondary data from 8582 MSM and 4163 TGH in Mumbai, India.

### Study site, study population, and procedures

These secondary data are from five targeted intervention programmes for MSM and TGH each under the aegis of Mumbai Districts AIDS Control Society (MDACS). The targeted interventions (TIs) are a part of the prevention component of the National AIDS Control Programme (NACP), and operational guidelines for TIs were prepared under NACP III.16 The main services under these TI programmes for high risk groups of MSM and TGH include outreach behaviour change communication (BCC), providing services (such as condoms, STI care, HIV testing), create an enabling environment, and community mobilisation.16 Each TI project is implemented by a non-government organization or community based organization. It has a registered population of about 650–2000 self-identified individuals (from the MSM and TGH groups) with high-risk sexual behaviour usually located in the local geography. The TI project has a team of trained outreach workers and peer educators from the same community to provide the outreach prevention services to the registered clients through regular personal contact. These outreach workers meet individuals who are a part of the TI regularly to discuss safe sex practices, distribute condoms, and to encourage them for clinical visits. The outreach workers are trained to collect sexual and behavioural data. The data on sexual activity, behaviours, and follow-up in the TI clinic for medical services are collected every three months for all registered individuals on a pre-designed format. Along with outreach services, this data collection activity is a part of regular TI program monitoring. The data collected every quarter are then shared with the MDACS for monitoring and planning of context specific interventions. Furthermore, the HIV status of these individuals are also recorded every six months. We used these existing data for the present secondary data analysis. The data used for the present analyses were collected by the outreach workers from all the individuals from these 10 TIs (five MSM and five TGH) from April through June 2020 (the behaviour patterns were for January through March 2020). The de-identified programmatic data of all the MSM and TGH from these 10 TIs were included for the present analysis. Usually, the quarterly data are collected by meeting these individuals in-person. However, due to COVID-19 travel restrictions during April through June 2020, the data for this quarter were collected telephonically.

We abstracted the following parameters for the present analysis: 1) Demographic data: age; gender; the main profession/job typology of the key population (KP)—this was classified as MSM or TGH for the present analysis; sub-typology—this variable was only for the MSM TIs and they were classified as *kothis* or *double deckers*. *Kothis* are “*males who show varying degrees of feminity*, *take the female role in sexual relationships with other men*, *and are involved mainly*—*though often not exclusively*—*in receptively anal/oral sex with men”* [page 12] and *“Kothis and hijras label those males who both insert and receive during penetrative sexual encounters (anal or oral) with other men as double deckers”* [Page 12] [16] (these are standard operational definitions for these sub-groups in MSM in the targeted intervention programmes in India; *kothis* and *double deckers* may be loosely considered equivalent to ‘bottom’ and ‘versatile’) 2) TI related data: years of association with the TI; 3) Internet apps used: number of online/mobile apps used, type of online/mobile apps; 4) Sexual behaviours: number of partners, number of partners from virtual space, number of partners from the non-virtual (physical) space; 5) Other risk behaviours: group sex (had sex with more than one sexual partner at the same time), attending group parties (many MSM/TGH may have multiple sexual encounters during these parties), mobility for sexual partners (travelled from usual place of residence/region to another district/region for sexual activity); 6) condom use: data collected as condom not used for at least one penetrative sex act in the past one week; and 7) HIV status.

### Statistical methods

We estimated the means and standard deviations (SDs) or median and interquartile ranges (IQR) for continuous variables. We estimated the proportions for categorical variables. The means between group were compared using the t-test and the medians were compared using the Mann Whitney Wilcoxon test. The proportions across groups were compared using the chi square test or Fishers’ exact test for low expected cell counts. Initially, we compared the behaviours between MSM and TGH, in addition to identifying the risk factors in each group. After the initial comparison, we compared these variables according to sub-typology (*kothis* and double deckers) in the MSM population.

We studied the association between number of online applications and high-risk behaviours (group sex, attending parties, and mobility for sex). We used logistic regression models to estimate the odds ratios (OR) and their 95% confidence intervals (CIs) as a measure of association. The primary explanatory variable was the number of applications and the additional variables in the multivariate variables were age, typology, and years of association with the TI. Finally, we used Venn diagrams to study the overlap patterns between virtual partners and these high-risk behaviours. We initially compared these patterns among MSM and TGH. We then compared these patterns in HIV negative and positive MSM, *kothis* and double decker MSM, and MSM according to their age groups. Only three data points were missing for five variables. This was about 0.02% of the total data; hence, we performed the analysis as it is (with the existing data). Data were analysed using Stata Version 15.1 (© StataCorp, College Station, Texas, USA).

The study was approved by the Institutional Ethics Committee of Mumbai Districts AIDS Control Society as a secondary data analysis (Ref No. 005/2021 Date: 27 October 2021).

## Results

### Demographic and mobile use data

The mean (SD) age of MSM was 31.1 (7.3) years and of TGH was 32.6 (6.5) years (p<0.001). Most of the MSM worked in the private sector (67%), about 8% were still in college, and 7% worked as masseurs. However, majority of the TGH were in sex work (86%) or *mangti* (29%) (asking for alms at traffic signals or other prominent locations). MSM were significantly more likely to have a mobile phone (88% vs 51%, p < 0.001) and internet access over the phone (78% vs 27%; p < 0.001) compared with TGH. The most common online/ phone-based app used for finding sexual partners by MSM was Grindr (48%), followed by Facebook (42%) and Blued (36%). The common online/apps used by TGH were Facebook (16%), Planet Romeo (14%) and Grindr (14%). In MSM, *double deckers* were significantly more likely to have a mobile phone (92% vs 82%; p < 0.001) and internet access over the phone (85% vs 70%; p < 0.001) compared with *kothis*. The common sites/apps used by these two sub-types differed. For instance, *double deckers* commonly used Grindr (59%), Blued (42%), and Facebook (42%); whereas *kothis* were more likely to use Facebook (42%), Grindr (36%), and Blued (28%). MSM were significantly more likely to have missed a condom at least once during the past one week compared with TGH (21.9% vs 3.9%; p<0.001). Details about these parameters are shown in Tables 1 and 2.

**Table 1:** Table comparing the demographics, internet practices, and high-risk behaviours of 8582 MSM and 4163 TGH, Mumbai, India.

	Total	MSM	TGH	P value
	N (%)	n (%)	n (%)	
Total	12,745 (100)	8582 (67.3)	4163 (32,7)	
Age [Mean (SD)]	31.6 (7.1)	31.1 (7.3)	32.6 (6.5)	<0.001
Association with targeted intervention programme (yrs) [Median (IQR)]	8 (3, 10)	8 (4, 9)	8 (2, 10)	<0.001
***Online/App related behaviours***				
Has a smart phone	9680 (75.9)	7546 (87.9)	2134 (51.3)	<0.001
Has mobile based internet	7848 (61.6)	6726 (78.4)	1122 (26.9)	<0.001
Applications/Web based sources				
Tinder	1129 (8.9)	837 (9.8)	292 (7.0)	<0.001
Grindr	4755 (37.3)	4191 (48.8)	564 (13.6)	<0.001
Blued	3657 (28.7)	3105 (36.2)	552 (13.3)	<0.001
Planet Romeo	2509 (19.7)	1929 (22.5)	580 (13.9)	<0.001
Facebook	4251 (33.4)	3585 (41.8)	666 (16.0)	<0.001
Had a virtual partner	10608 (83.2)	7833 (91.3)	2775 (66.7)	<0.001
***Last 10 partners***				
Partners met in physical space [Mean (SD)]	6.3 (2.6)	5.7 (2.5)	7.5 (2.3)	<0.001
Partners met in virtual space [Mean (SD)]	3.7 (2.6)	4.3 (2.5)	2.5 (2.3)	<0.001
***Risk behaviours***				
Attended Parties	2465 (19.3)	2365 (27.6)	100 (2.4)	<0.001
Had group sex	1622 (12.7)	1365 (15.9)	257 (6.2)	<0.001
Mobility for sex	2274 (17.8)	2112 (24.6)	162 (3.9)	<0.001
Mobility days (Median SD])	3 (2, 4)	3 (2, 4)	2 (2, 3)	<0.001
***Condom use***				
Missed condom at least once ^a^	2047 (16.1)	1882 (21.9)	165 (3.9)	<0.001
***HIV infected***	263 (2.1)	153 (1.8)	110 (2.6)	0.001

^a^ Missed condom at least once during penetrative sex in the past one week

**Table 2:** Table comparing the demographics, internet practices, and high-risk behaviours of among different types of MSM in Mumbai, India.

	Total	Kothi	Double decker	P value
Total	8582 (100)	3706 (43.2)	4876 (56.8)	
Age [Mean (SD)]	31.1 (7.3)	30.7 (7.5)	31.4 (7.1)	<0.001
Association with targeted intervention programme (yrs) [Median (IQR)]	8 (4, 9)	6 (4, 9)	9 (4, 10)	<0.001
***Online/App related behaviours***				
Has a smart phone	7546 (87.9)	3055 (82.4)	4491 (92.1)	<0.001
Has mobile based internet	6726 (78.4)	2592 (69.9)	4134 (84.8)	<0.001
Applications/Web based sources				
Tinder	837 (9.8)	140 (3.8)	697 (14.3)	<0.001
Grindr	4191 (48.8)	1335 (36.0)	2856 (58.6)	<0.001
Blued	3105 (36.2)	1044 (28.2)	2061 (42.3)	<0.001
Planet Romeo	1929 (22.5)	654 (17.7)	1275 (26.2)	<0.001
Facebook	3585 (41.8)	1545 (41.7)	2040 (41.8)	0.89
Had a virtual partner	7833 (91.3)	3277 (88.4)	4556 (93.4)	<0.001
***Last 10 partners***				
Partners met in physical space [Mean (SD)]	5.7 (2.6)	6.4 (2.3)	5.2 (2.6)	<0.001
Partners met in virtual space [Mean (SD)]	4.3 (2.5)	3.6 (2.3)	4.8 (2.6)	<0.001
***Risk behaviours***				
Attended Parties	2365 (27.6)	887 (23.9)	1478 (30.3)	<0.001
Had group sex	1365 (15.9)	504 (13.6)	861 (17.7)	<0.001
Mobile for sex	2112 (24.6)	1063 (28.7)	1049 (21.5)	<0.001
Mobility days (Median SD])	3 (2, 4)	3 (2, 4)	3 (2, 4)	<0.001
***Condom use***				
Missed condom at least once ^a^	1882 (21.9)	1189 (32.1)	693 (14.2)	<0.001
***HIV infected***	153 (1.8)	65 (1.8)	88 (1.8)	0.86

^a^ Missed condom at least once during penetrative sex in the past one week

**Fig 1:**
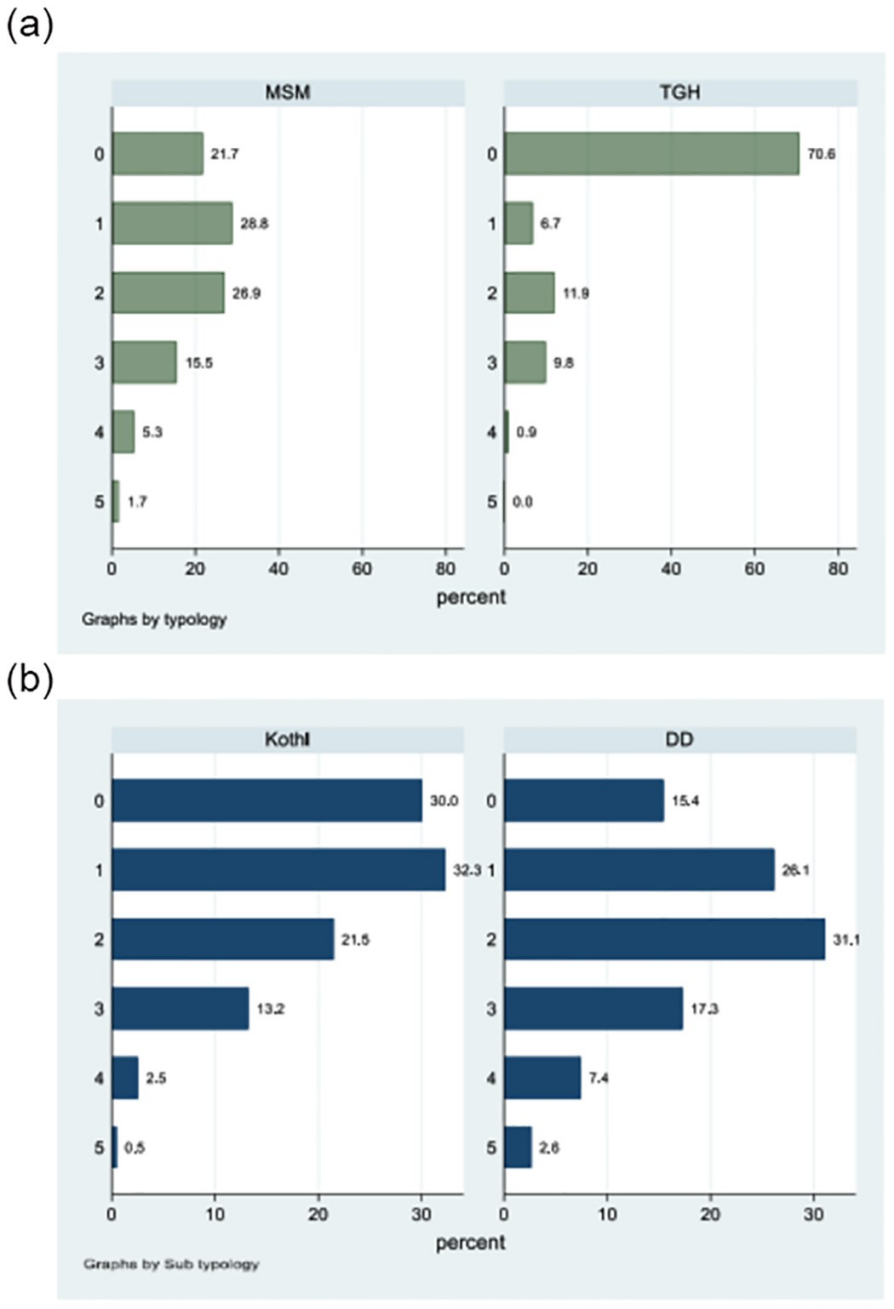
a. Figure showing the number of web-based/online applications by men who have sex with men and male-to-female transgendered people, Mumbai, India. b. Figure showing the number of web-based/online applications by *kothis* and double- deckers, 8592 men who have sex with men in Mumbai, India.

About 22% of MSM did not use any online/internet-based app compared with 71% of TGH (p < 0.001). The median (IQR) number of sites that were used by MSM (1, [1, 2]) was significantly higher compared with TGH (0, [0.1]) (p<0.001). Among MSM, 15% of *double deckers* did not use any online/internet-based sites/apps compared with 30% of *kothis* (p < 0.001). The median (IQR) of sites used by *double deckers* (2, [2, 3]) was significantly higher compared with *kothis* (1, [1, 2]) (p < 0.001). MSM and TGH in the 18–24-year age group were most likely to use these applications. Details about the number of sites/apps used according to the typology (MSM or TGH) and sub-typology (*kothis* and *double* deckers) are presented in Fig 1a and 1b.

### Sexual behaviours

MSM were significantly more likely to have had a partner from the virtual space compared with TGH (91% vs 67%; p < 0.001). The mean (SD) number of partners (among the last 10 partners) found at physical space was significantly higher in TGH compared with MSM (7.5 [2.3] vs 5.7 [2.5]; p < 0.001). MSM were more likely to have attended parties (28% vs 2%; p < 0.001), had group sex (16% vs 6%; p < 0.001), and were mobile for sex (25% vs 4%). Among MSM, *double deckers* were more likely to have a partner from virtual sites compared with *kothis* (93% vs 88%, p < 0.001). HIV positive MSM (Median: 4, IQR: 3, 8) had a significantly higher number of partners from the virtual space compared with HIV negative MSM (Median: 4, IQR: 2, 6). Even though *double deckers* were more likely to attend parties (30% vs 24%; p < 0.001) and had group sex (18% vs 14%; p < 0.001), they were less likely to be mobile for sexual activities (22% vs 29%; p < 0.001) compared with *kothis*. In general, reporting ‘missed a condom at least once during penetrative sex act in the past one week’ was significantly higher among those who had a partner from the virtual space compared with those who did not (16.9% vs 12.0%; p<0.001). TGH who reported group sex were significantly more likely to have missed condom use at least once in the past one week (7.8% vs 3.7%; p<0.001). MSM and TGH who reported attending parties were significantly less likely to report ‘missed condom use at least once in the past one week (14.2% vs 16.5%; p = 0.006). MSM and TGH who were mobile for sex were significantly more likely to report ‘missed condom use at least once’ (29.7% vs 13.1%; p<0.001). Details about these behaviours are presented in Tables 1 and 2.

### Multivariate analysis

In the logistic regression models, we found that TGH were significantly less likely to have had group sex, attended parties, or moved from the site of residence for sex. After adjusting for typology, age, and years of association with the TI programme, we found that those who use three or more apps/online platforms were significantly more likely to attend parties (OR: 7.13, 95% CI: 5.97, 8.51; p < 0.001), have group sex (OR: 2.88, 95% CI: 2.46, 3.37; p < 0.001), and were mobile for sex (OR: 11.82, 95% CI: 9.99, 13.99; p < 0.001). We also found that MSM and TGH in the older age groups were less likely to attend parties or have group sex, but were more likely to move from the usual place of residence for sex (Table 3).

**Table 3:** Logistic regression models showing the factors associated with risk behaviours in 8582 MSM and 4163 TGH, Mumbai, India.

	Attended parties	Reported Group Sex	Was mobile for sex
No. of online platforms			
None	Reference	Reference	Reference
Upto 2	4.95 (4.20, 5.83) ***	1.48 (1.28, 1.72) ***	2.69 (2.29, 3.16) ***
3 or more	7.13 (5.97, 8.51) ***	2.88 (2.46, 3.37) ***	11.82 (9.99, 13.99) ***
Typology			
MSM	Reference	Reference	Reference
TGH	0.12 (0.10, 0.15) ***	0.47 (0.41, 0.55) ***	0.15 (0.13, 0.18) ***
Age groups			
18–24	Reference	Reference	Reference
25–29	1.26 (1.08, 1.47) **	0.99 (0.83, 1.19)	0.92 (0.78, 1.10)
30–34	0.97 (0.81, 1.17)	0.79 (0.64, 0.96) *	1.44 (1.19, 1.74) ***
35–39	0.79 (0.64, 0.98) *	0.60 (0.47, 0.76) ***	1.52 (1.21, 1.91) ***
> = 40	0.76 (0.61, 0.96) *	0.65 (0.50, 0.83) **	1.53 (1.22, 1.94) ***
Association with the current targeted intervention (in years)			
0–5	Reference	Reference	Reference
6–10	1.01 (0.87, 1.18)	1.40 (1.19, 1.64) ***	0.91 (0.77, 1.07)
> = 11	1.01 (0.84, 1.23)	1.07 (0.86, 1.33)	0.12 (0.10, 0.16) ***

Among MSM, we found that those in the older age groups were significantly less likely to use these apps or have had a partner from the virtual space (Table 4). Furthermore, *double deckers* were significantly more likely to have used these apps (OR: 2.23, 95% CI: 1.99, 2.50; p < 0.001) and had found a partner in the virtual space (OR: 1.81, 95% CI: 1.54, 2.13; p < 0.001). We found *double deckers* were more likely to attend parties (OR: 1.13, 95% CI: 1.02, 1.26; p = 0.02) and had group sex (OR: 1.19, 95% CI: 1.04, 1.35; p = 0.01), but were significantly less likely to be mobile for sex (OR: 0.55, 95% CI: 0.49, 0.72; p < 0.001). After adjusting for age, sub-typology, and years of association with the TI, we found that those who used three or more apps/online were significantly more likely to have these three risk behaviours (Table 5). In multivariate analysis for condom use, we found that MSM & TGH TI population who reported mobility for sex were significantly more likely to have missed a condom in the past one week (OR: 2.79, 95% CI: 2.46, 3.16; p<0.001). However, MSM and TGH who were associated with TI for six to 10 years (OR: 0.47, 95% CI: 0.40, 0.55) and more than 10 years (OR: 0.37, 95% CI: 0.30, 0.46; p<0.001) were significantly less likely to report ‘missed condom at least once during penetrative sex acts in the past one week.

**Table 4:** Logistic regression models for “use of online/mobile apps” and “having a partner from the virtual space” among 8582 men have sex with men, Mumbai, India.

	Use of internet based/ online apps	Has a partner from the virtual space
Age groups		
18–24	Reference	Reference
25–29	0.49 (0.42, 0.58) ***	0.62 (0.47, 0.81) ***
30–34	0.53 (0.43, 0.65) ***	0.50 (0.37, 0.67) ***
35–39	0.52 (0.41, 0.66) ***	0.66 (0.46, 0.95) *
> = 40	0.47 (0.37, 0.60) ***	0.54 (0.37, 0.77) **
Typology		
*Kothi*	Reference	Reference
Double decker	2.23 (1.99, 2.50) ***	1.81 (1.54, 2.13) ***
Association with the current targeted intervention (in years)		
0–5	Reference	Reference
6–10	2.18 (1.86, 2.56) ***	0.92 (0.73, 1.15)
> = 11	1.71 (1.37, 2.13) ***	1.29 (0.93, 1.79)

**Table 5:** Logistic regression models for risk behaviours (attended parties, reported group sex, and were mobile for sex) among 8582 men have sex with men, Mumbai, India.

	Attended parties	Reported Group Sex	Was mobile for sex
No. of online platforms			
None	Reference	Reference	Reference
Upto 2	4.38 (3.68, 5.21) ***	1.90 (1.58, 2.29) ***	3.07 (2.55, 3.71) ***
3 or more	6.80 (5.63, 8.21) ***	3.81 (3.12, 4.65) ***	19.10 (15.59, 23.40) ***
Typology			
*Kothi*	Reference	Reference	Reference
*Double decker*	1.13 (1.02, 1.26) *	1.19 (1.04, 1.35) **	0.55 (0.49, 0.62) ***
Age groups			
18–24	Reference	Reference	Reference
25–29	1.19 (1.01, 1.40) *	1.27 (1.05, 1.54) *	0.75 (0.62, 0.90) **
30–34	0.86 (0.71, 1.05)	0.94 (0.74, 1.18)	1.04 (0.84, 1.29)
35–39	0.69 (0.55, 0.86) **	0.66 (0.49, 0.87) **	1.11 (0.86, 1.43)
> = 40	0.65 (0.52, 0.83) ***	0.73 (0.55, 0.97) *	1.08 (0.83, 1.40)
Association with the current targeted intervention (in years)			
0–5	Reference	Reference	Reference
6–10	1.17 (0.99, 1.37)	1.09 (0.90, 1.32)	1.44 (1.21, 1.73) ***
> = 11	1.12 (0.90, 1.38)	0.99 (0.77, 1.27)	0.21 (0.15, 0.28) ***

### Venn diagrams

**Fig 2:**
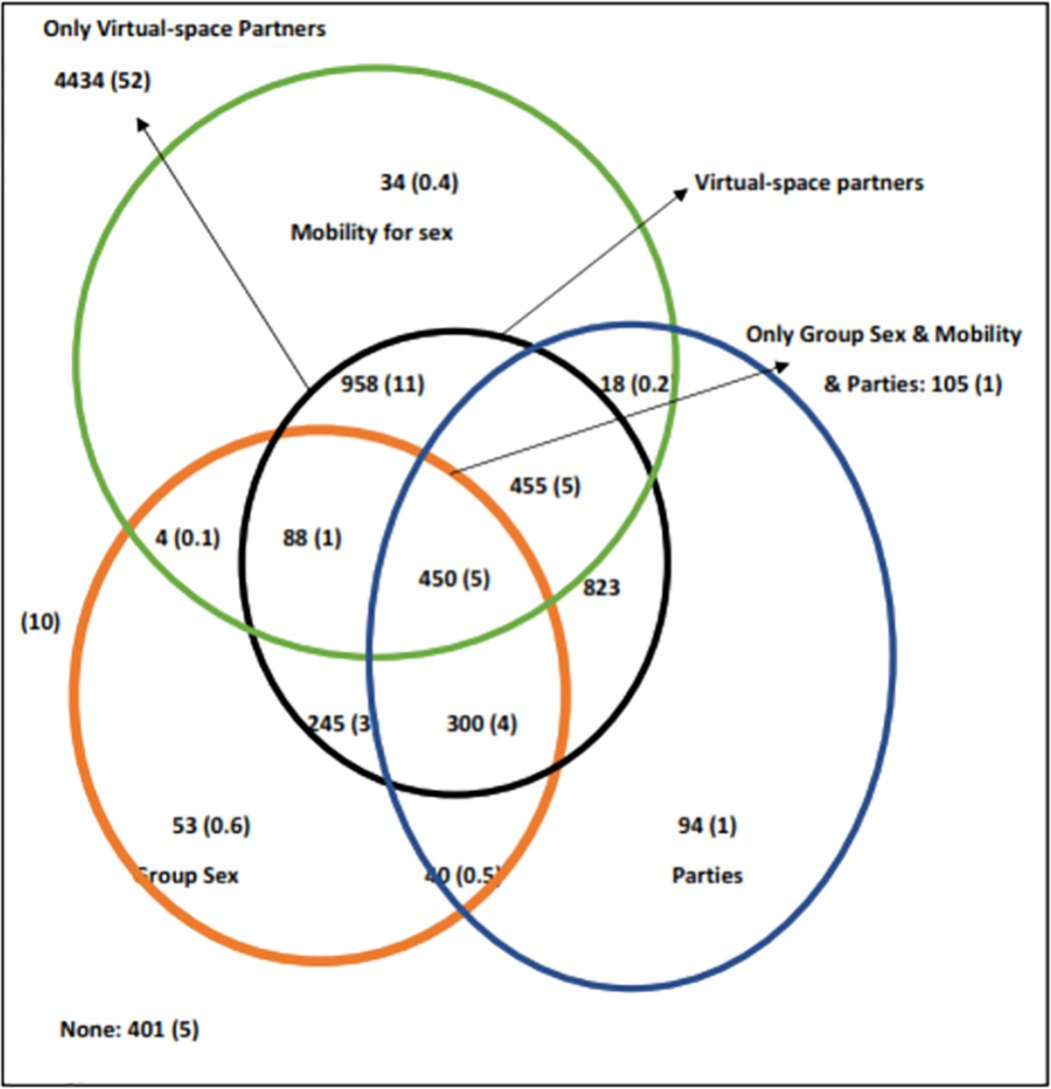
Venn diagram showing the distribution and overlap of various behaviours (partners from virtual space, group sex, mobility for sex, attend parties) in 8582 men who have sex with men in Mumbai, India.

**Fig 3:**
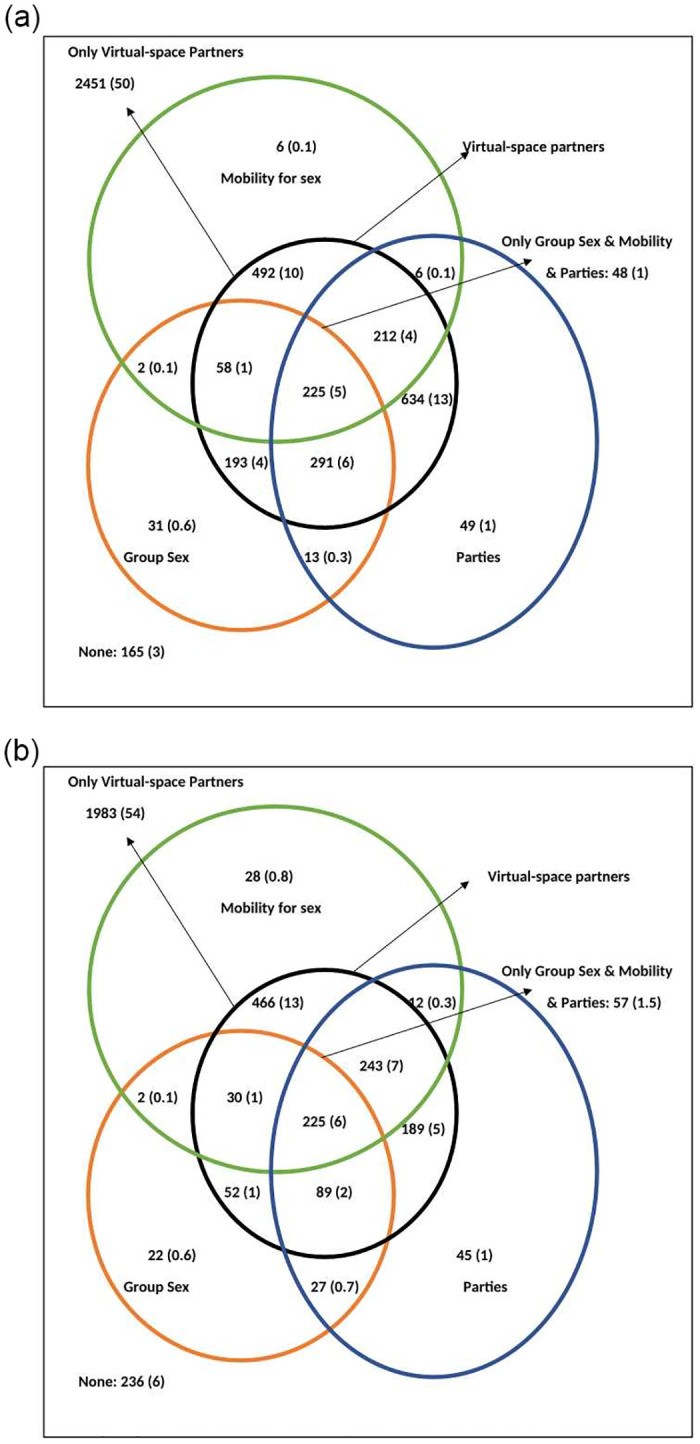
a. Venn diagram showing the distribution and overlap of various behaviours (partners from virtual space, group sex, mobility for sex, attend parties) in men who have sex with men who identify as double-deckers (DDs), Mumbai, India. b. Venn diagram showing the distribution and overlap of various behaviours (partners from virtual space, group sex, mobility for sex, attend parties) in men who have sex with men who identify as *kothis*, Mumbai, India.

**Fig 4:**
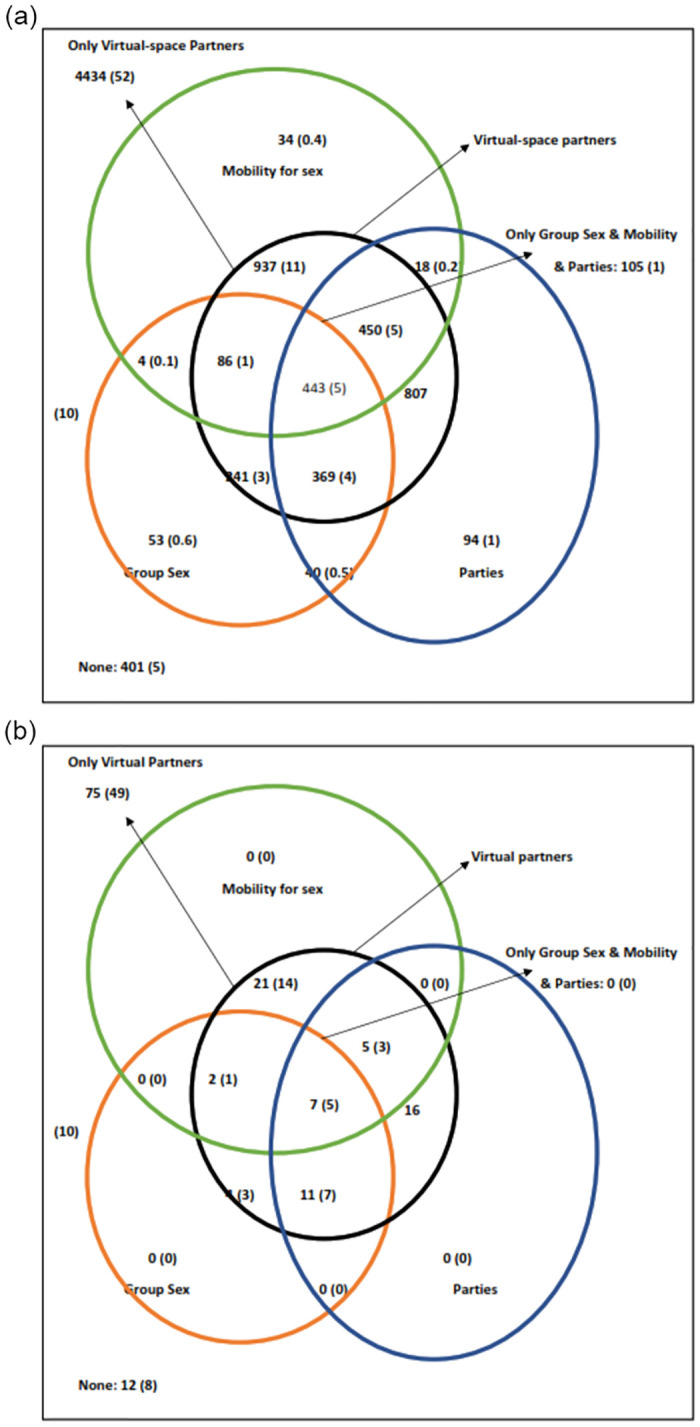
a. Venn diagram showing the distribution and overlap of various behaviours (partners from virtual space, group sex, mobility for sex, attend parties) in HIV negative men who have sex with men, Mumbai, India. b. Venn diagram showing the distribution and overlap of various behaviours (partners from virtual space, group sex, mobility for sex, attend parties) in HIV infected men who have sex with men, Mumbai, India.

When we drew Venn diagrams (Figs 2–4) to understand the overlap of behaviours in MSM, we found that in HIV positive MSM, all the three risk behaviours (group sex, parties, and mobility for sex) were in those who reported partners from the virtual space. All, but two of these MSM were detected HIV positive before data collection period; thus, they were aware of their status during this period. In HIV negative MSM, however, these behaviours were also reported in those who did not have partners from the virtual space (Fig 4a and 4b). Thus, among the group of MSM who had partners from physical space, HIV negative MSM were significantly more likely to have any of these three risk behaviours (group sex, parties, and mobility for sex) compared with HIV positive MSM (47.2% vs 0%, p = 0.001). We also found that the proportion of any of these three risk behaviours (group sex, parties, and mobility for sex) and overlap of virtual partners was highest in age group of 35–39 years (42.7%) and those 40 years of age and above (42.6%) compared with the younger age groups (p < 0.001). Finally, when we compared *kothis* and *double deckers*, we found that proportion of any of these three risk behaviours and overlap of virtual partners was significantly higher in *double deckers* compared with *kothis* (43.2% vs 34.9%, p < 0.001). However, *kothis* were significantly more likely to have all these three behaviours and partners from the virtual space compared with *double deckers* (6.1% vs 4.6%, p = 0.003). Distribution and overlap of these behaviours are presented in Figs 2–4 (Fig 2: MSM population; Fig 3: according to sub typology [*kothis &* double deckers]; Fig 4: according to HIV status [HIV negative and positive]).

## Discussion

Thus, we found that internet-based applications were more commonly used by MSM compared with TGH. Among MSM, *double deckers* were more likely to use these applications compared with *kothis*. In general, higher number of internet-based sites/app use was associated with attending parties, reporting group sex, or being mobile for sex partners. MSM in the age group of 20–25 years were more likely to attend parties and report group sex, however, they were less likely to be mobile for sex. There was complete overlap between HIV positive MSM reporting risky behaviours (attending parties, group sex, and mobile for sex) and those who found partners in the virtual space. MSM and TGH who reported partners from the virtual space were significantly more likely to have missed condom use at least once during the past one week.

As observed in our data, there was a distinct difference in the pattern of internet use and internet-based site/application use between MSM and TGH. In general, TGH were less likely to own a smart phone, have access to internet on phone, and use these apps. This may be due to the difference in the socio-economic status of these two groups. TGH have been a marginalised community in India; they may not complete formal education and may end up working in the informal economy [17–22]. In contrast, majority of the MSM were employed in formal structure, and a high proportion of them used smart phones and had access to internet on these phones. Furthermore, TGH had a significantly lower number of partners from the virtual space. They were also less likely to report risk-behaviours such as group sex or mobility for sex compared with MSM. Even though the virtual space may be a safe space for trans-women, they may be the subject of harassment and abuse by the individuals including some from the larger LGBTQ community and social circle [23]. Perhaps, TGH were less comfortable in seeking partners online to avoid rejection, harassment, or stigmatization. They may be more comfortable in the traditional cruising spots or other physical spots where they solicit partners or clients. At present there is a lot of emphasis on internet-based interventions and researchers have suggested the use of these interventions in MSM and TGH [24]. However, given the low penetration of the mobile-based internet in the TGH population who are a part of targeted interventions, we would recommend physical targeted interventions in this population. Internet-based interventions, however, can be actively encouraged for MSM even among TIs.

Another interesting observation was the difference in the risk behaviours between HIV positive and HIV negative MSM. As seen in our data, there was a complete overlap between those who had partners from the virtual space and those reporting risky sexual behaviours (such as group sex, attending parties, or being mobile for sexual activity) in HIV positive MSM. However, among HIV negative MSM, these risk behaviours were also observed in those who did not have partners from the virtual space. The online space provides anonymity to MSM—they can remain anonymous about their sexual orientation as well as their HIV status. For instance, Esktrand and co-workers [25]—in an on-line survey of MSM—found that men who identified themselves are ‘bisexual’ are not out to others, married, and present themselves as ‘heterosexual’ in the community, and have unprotected anal sex. Another study by Rhoton and colleagues [26] found that even though MSM who use these dating apps may have unprotected anal sex, they are less likely to disclose their HIV status to their partners. Studies have found that many HIV positive MSM may not disclose their status in their online profiles due to stigma and fear of rejection by potential partners [27–32]. Furthermore, it is also reported that LGBTQ may use these online apps to express their identities and find partners, and protect themselves from stigmatization [33]. Thus, the online space allows MSM to find sexual partners without disclosing a lot of personal information. This route may be preferred by those who are closeted and do not identify themselves as ‘gay’, ‘homosexual’, or who may not want to disclose their HIV status. In the physical space, such as cruising places (parks, public toilets, other public areas), there is always a chance that these individuals may encounter someone else from the community who may be aware of their HIV status (they may have visited the same clinic or testing centre). Thus, there is a fear that their status may be known to others in the community which may lead to rejection by partners. The online space, however, provides anonymity and choice to interact with specific community members while avoiding others. Many MSM, who access partners through the virtual space and wish to remain anonymous may not be willing to become a part of the existing targeted interventions and avail services at drop-in centres. Given these findings, internet-based interventions [15]—particularly targeted towards HIV infected MSM should be a major component of the National AIDS Control Programme and it should be included in all targeted interventions. This may require additional training of existing outreach workers in internet outreach; thus, guidelines and protocols should be developed for these interventions.

As observed in our data, MSM were more likely to report risky behaviours such as group sex, attending parties, or being mobile for sex. MSM are mobile not only across districts but also across states in India; these highly mobile MSM were more likely to be associated with unsafe sexual practices and were more likely to be HIV infected [34]. Group sex—another high-risk behaviour found in our study—was associated with a higher number of internet-based app/online apps used; a feature also found by other authors [35]. Many MSM attend these weekend parties, where along with dance, there may be alcohol, drug use, and unprotected sex—sometimes with multiple partners in the same party [36–39]. Interventions at these parties may require specific techniques and messaging [40, 41]. Thus, it is important to understand the behaviour of MSM who attend parties (probably through qualitative and mixed-method studies) and potential risk-reduction strategies which will be acceptable by this population. There is a need to regularly monitor these risk behaviours in the TI programmes. MSM and TGH who reported partners from the virtual space, and those who were mobile for sex were significantly more likely to report ‘having missed a condom at least once during penetrative sex acts in the past one week’. Thus, intervention strategies for risk-reduction and consistent condom use in MSM who were mobile for sex, those who have group sex, and those who attend parties should be included in future national programmes in India.

The study was not without limitations. We did not have information on the type of partner with whom they had group sex (partners who were found on the virtual space or physical space) or whether condoms were missed during sex with these partners. This was not a detailed behavioural survey but a programmatic assessment of internet-related behaviours in core high-risk groups who have been a part of intervention programmes over the past few years. However, we used the Venn diagrams to study the overlap of behaviours in these groups and sub-groups. The diagrams give useful information on grouping of behaviours in MSM and TGH, and in various sub-groups of MSM. We also did not have information on other biological indicators (such as presence of STIs other than HIV).

Nonetheless, the study provides useful information for modification of the existing TIs for high-risk groups in India. Younger MSM, particularly those who identified as ‘*double deckers*’ were more likely to use internet-based/online apps and have partners from the virtual space. Thus, internet-based interventions for MSM should be designed and incorporated in the existing programme and outreach workers should be trained for virtual outreach services. Most of the HIV infected MSM reported either one of these risk behaviours—group sex or attending parties, and being mobile for sex work. All these also reported having a partner from the virtual space. Therefore, messages specifically targeted towards HIV positive MSM about safe sex practices, risk reduction strategies, and treatment initiation and adherence should also be included in these online interventions. However, given the reach and use of smartphones and these apps among TGH, internet-based interventions may not be such a useful option for them, and the existing physical outreach in TIs should be strengthened. Data on high-risk behaviours such as group sex, attending parties, and mobility for sex should be collected as a part of regular monitoring in TI; and appropriate interventions should be designed and implemented for these individuals. Though online interventions may start in the metro cities; given, the speed of digitization and penetration of internet in India, these may be required in Tier 2 and Tier 3 cities as well. Thus, developing guidelines and standard operating procedures for online intervention strategies should be a priority for the national AIDS control programme in India.
